# Asking the right questions: developing evidence-based strategies for treating HIV in women and children

**DOI:** 10.1186/1471-2458-11-388

**Published:** 2011-05-25

**Authors:** Quarraisha Abdool Karim, Anchilla Banegura, Pedro Cahn, Celia DC Christie, Robert Dintruff, Manuel Distel, Catherine Hankins, Nicholas Hellmann, Elly Katabira, Sandra Lehrman, Julio Montaner, Scott Purdon, James F Rooney, Robin Wood, Shirin Heidari

**Affiliations:** 1 Department of Epidemiology, Columbia University, New York, USA; 2 Prevention and Epidemiology, Centre for the AIDS Programme of Research in South Africa, Durban, South Africa; 3 Global Virology Access, Tibotec Pharmaceuticals Ltd, Nairobi, Kenya; 4 Direccion Cientifica, Fundacion Huesped, Buenos Aires, Argentina; 5 Department of Pediatrics, University of the West Indies, Kingston, Jamaica; 6 Commercial Development, Abbott, Abbott Park, Illinois, USA; 7 Medical Affairs, Boehringer Ingelheim GmbH, Ingelheim, Germany; 8 Office of the Deputy Executive Director, UNAIDS, Geneva, Switzerland; 9 Medical and Scientific Affairs, Elizabeth Glaser Pediatric AIDS Foundation, Washington, DC, USA; 10 Department of Research, Makerere Medical School, Kampala, Uganda; 11 Scientific Affairs - Infectious Diseases, Office of the Chief Medical Officer, Merck & Co, Upper Gwynedd, Pennsylvania, USA; 12 AIDS Research and Head of Division of AIDS, University of British Columbia, Vancouver, Canada; 13 Access and Government Affairs, ViiV Healthcare, Middlesex, UK; 14 Medical Affairs, Gilead Sciences, Foster City, California, USA; 15 Institute of Infectious Disease and Molecular Medicine, Desmond Tutu HIV Centre, University of Cape Town, South Africa; 16 Research Promotion, International AIDS Society, Geneva, Switzerland

## Abstract

In July 2010, the World Health Organization (WHO) issued formal revisions of its guidelines on the use of highly active antiretroviral therapy for HIV. The new guidelines greatly expand eligibility for treatment of adults and children, as well as for pregnant women seeking prophylaxis for vertical HIV transmission. WHO's new recommendations bring the guidelines closer to practices in developed countries, and its shift to earlier treatment alone will increase the number of treatment-eligible people by 50% or more.

Scaling up access to HIV treatment is revealing important gaps in our understanding of how best to provide for all those in need. This knowledge gap is especially significant in developing countries, where women and children comprise a majority of those living with HIV infection. Given the magnitude and significance of these populations, the International AIDS Society, through its Industry Liaison Forum, prioritized HIV treatment and prophylaxis of women and children. In March 2010, the International AIDS Society and 15 partners launched a Consensus Statement outlining priority areas in which a relative lack of knowledge impedes delivery of optimal prevention of mother to child transmission (PMTCT) and treatment to women and children.

The Consensus Statement, "Asking the Right Questions: Advancing an HIV Research Agenda for Women and Children", makes a special appeal for a more gender-sensitive approach to HIV research at all stages, from conception to design and implementation. It particularly emphasizes research to enhance the understanding of sex-based differences and paediatric needs in treatment uptake and response. In addition to clinical issues, the statement focuses on programmatic research that facilitates access and adherence to antiretroviral regimens. Better coordination of HIV management with sexual and reproductive healthcare delivery is one such approach.

We discuss here our knowledge gaps concerning effective, safe PMTCT and treatment for women and children in light of the expansion envisioned by WHO's revised guidelines. The guideline's new goals present an opportunity for advancing the women and children's agenda outlined in the Consensus Statement.

## Commentary

In July 2010, the World Health Organization (WHO) issued formal revisions of its guidelines on the use of highly active antiretroviral therapy for HIV. The guidelines greatly expand eligibility for treatment of adults [1] and children [2]. They also broaden the armamentarium for prevention of mother to child transmission (PMTCT) of HIV [3]. WHO's recommendations, which apply mainly to resource-restricted settings, bring the guidelines closer to the practices in developed countries [4]. At the same time, they will increase the number of treatment-eligible patients by 50% or more [5].

Changes in HIV treatment access are of special concern to women and children. Women notably constitute 60% of the people living with HIV in the high-burden countries of sub-Saharan Africa [6]. Furthermore, expectant HIV-infected mothers face the combined hazard of virus- and pregnancy-related health issues; they have maternal mortality rates that are as much as 10-fold higher than their HIV-uninfected peers [7]. This excess mortality might seem self-evident, yet research has yet to break down the various contributing factors, including AIDS or other comorbidities and obstetric conditions aggravated by immune deficiency. Children in the least developed countries, meanwhile, suffer from the maternal HIV epidemic, as well as the paediatric one, leading to enhancement of a third concurrent epidemic, malnutrition.

Considering the need to optimize the benefits of antiretroviral treatment for women and children, the International AIDS Society's Industry Liaison Forum (IAS-ILF) launched a mapping exercise to examine the need for further clinical and operations research related to HIV treatment and PMTCT [8]. The mapping exercise resulted in the Consensus Statement, "Asking the Right Questions: Advancing an HIV Research Agenda for Women and Children", which was released on 8 March 2010 by the IAS and 15 partners, including United Nations agencies, community groups, the private sector and foundations [9]. The statement develops a useful roadmap for developing the means to expand optimum HIV care for women and children in the manner envisioned by WHO's 2010 HIV guidelines. We discuss here in detail the missing pieces in our knowledge of optimal HIV treatment in women and children and advocate advancing this research agenda.

### Overcoming the hurdles to treating HIV in women

The WHO guidelines advance the CD4 count threshold for initiating treatment in patients older than five years to 350 cells/mm^3 ^from the previous limit of 200 cells/mm^3 ^[1]. All patients with clinically advanced HIV (WHO stages 3 and 4) are also eligible.

These criteria hold for women, as well as for men. Yet an old but unresolved debate is whether there are biological differences between men and women that influence disease progression and treatment outcome. Two studies, each enrolling more than 2000 participants, have reported that women on antiretroviral (ARV) drugs have higher CD4 counts and slower disease progression than men [10,11]. Other studies have not observed any differences [12,13]. Resolution of this issue is made difficult by short follow up and many confounding factors, such as concurrent health problems, care access, ethnic origin and socioeconomic factors.

Another area of uncertainty over sex differences concerns pharmacokinetics and toxicities. These issues arise for nevirapine and efavirenz, the two drugs that provide the high potency of WHO's preferred first-line combination regimens. There are long-established sex differences in nevirapine's adverse effects: women with higher CD4 counts are more at risk than men at the same CD4 count for hypersensitivity reactions and hepatic failure after starting the drug [14,15]. Recent studies have contested this CD4 association, however [16,17]. An analysis of efavirenz found equal virologic and immunologic improvements in men and women, but more frequent toxicity-related drug discontinuations in women [18]. In treatment outcome studies, factors such as race, lower body weight or liver function differences may confound the results.

A third major area in which sex can complicate treatment decisions is hormonal contraceptives. Although there is as yet no evidence that hormonal contraception interferes with ARVs, there is evidence that the reverse is true. Women receiving nevirapine- or ritonavir-containing regimens (which are favoured by WHO for second-line therapy) will more rapidly metabolize ethinyl oestradiol and norethindrone taken for contraceptive purposes. Progesterone-based contraceptives may still be feasible, however [19].

There are also controversies over the effect of hormonal contraception on HIV progression. Some published data suggest that hormonal contraception increases the risk of HIV acquisition [20] or disease progression [21], whereas other studies contradict these results [22,23]. There is an urgent need to conclusively resolve this question.

In addition, there has been little investigation of the interaction between ARVs and the female hormone changes that women experience in the course of their lives. A number of studies have found that pregnancy reduces antiretroviral drug levels [24]. There are also indications that some women on ARVs undergo earlier onset of menopause [25].

In contrast, the relationship between ARVs and puberty is uncharted territory. It is possible, for example, that ARVs contribute to delaying puberty even though they reverse the endocrine-disrupting effects of chronic HIV [26]. Once teenagers do achieve puberty, optimal HIV treatment may be difficult to achieve because pubescent adolescents have unexpectedly low drug levels despite reasonable adherence [27] and are at increased risk of ARV-associated metabolic dysfunction [28]. With the growing availability of paediatric antiretroviral treatment, an increasing number of children are surviving into and through adolescence. The long overlooked research on this age group's response to ARVs has become a pressing issue.

Women are in a more vulnerable position than men in many parts of the world, and often their lesser socioeconomic status and decision-making power constrains their ability to seek out care on their own [29-31]. Nonetheless, a greater proportion of women than men with HIV are diagnosed and receive ARVs. Due to their biologic, social and family roles, women more readily seek contact with the healthcare system, where they can receive HIV testing [32-34] and care [34-36].

Women would benefit, therefore, from an integration of HIV programmes with broader sexual and reproductive health services [37,38]. This possibility signals the need to intensify oft-ignored operations research into women's care delivery. Such research, which includes programme evaluations, can show how to optimize antiretroviral therapy by improving HIV diagnosis and long-term follow up of women at different socioeconomic levels. The need is particularly critical for marginalized subpopulations of adolescent and adult women. Women living in remote areas, single women, widows, sex workers, women who use drugs, transgender individuals and women who are indigent or from racial minorities should receive special attention.

### Extending prophylaxis for prevention of vertical transmission

In 2008, an estimated 45% of pregnant women in resource-limited countries received PMTCT regimens, but these were generally simpler and shorter than the regimens that WHO now advocates [39].

The 2010 PMTCT guidelines primarily advise provision of potent triple antiretroviral therapy for prevention of vertical transmission in pregnant women [3]. Thus, pregnant women who present at prenatal clinics with serious HIV-related symptoms or CD4 counts below 350 cells/mm^3 ^will receive a highly suppressive antiretroviral regimen, as recommended for all adults for their own health. The triple regimen will also minimize the risk of transmission of HIV to the foetus and breastfeeding infant.

The new WHO guidelines propose that expectant HIV-positive mothers with CD4 counts above 350 cells/mm^3 ^receive triple-drug regimens, too, starting at 14 weeks of gestation and continuing through weaning [3]. Evidence shows that suppressive combination therapy from late pregnancy through six months of breastfeeding can reduce mother to child HIV transmission to below 1% [40].

The guidelines, in addition, offer a cheaper option for women with CD4 counts above the 350 cells/mm^3 ^threshold: of zidovudine monotherapy from 14 weeks of gestation until birth. Mothers may also receive single-dose nevirapine during labour, plus a week of zidovudine/lamivudine combination post-partum. Breastfeeding infants in this case receive nevirapine prophylaxis until weaned. Non-breastfeeding infants receive nevirapine or zidovudine for six weeks after birth.

Studies that examine and compare the efficacy of WHO's two PMTCT options, triple regimen versus simplified regimen, would be very valuable. An 805-birth study in three African countries found no significant difference in pre-birth transmission rates when comparing three-drug regimens to what is now the simpler WHO alternative (though starting only after week 26) [41]. As for transmission during breastfeeding, a trial following 2369 Malawi births reported no significant difference between maternal triple therapy and infant daily nevirapine [42].

Another outstanding question is the impact of intermittent therapy on women's future treatment options. There is growing evidence that administering single-dose nevirapine during labour - the most common PMTCT regimen used in low-income countries - increases the risk of developing nevirapine-resistant HIV [43]. The revised guidelines recommend seven days of zidovudine/lamivudine post-partum to overcome this problem by inhibiting HIV replication while the nevirapine is slowly being cleared from the mother's body. Additional studies are needed to address resistance concerns of the guideline revisions, in particular, consequences of zidovudine monotherapy from 14 weeks of gestation and continued during pregnancy [44].

Furthermore, the Strategies for Management of Antiretroviral Therapy (SMART) trial found that episodic HIV-suppressive ARV combinations result in greater mortality and morbidity than continuous therapy [45,46]. Initial research has not confirmed this increase in women who terminate ARVs after delivery, but definitive data for women with CD4 counts of more than 350 cells/mm^3 ^are not yet available [47].

WHO's PMTCT revisions raise a number of operational issues concerning the ability of treatment programmes to accommodate the new recommendations. Initial studies indicate that instituting the new recommendations would triple the number of HIV infections averted [48,49]. Having effective regimens is not enough all by itself, however; effective delivery programmes are even more important [50].

There remain many research questions involving delivery of extended PMTCT [9]. Central to this effort is, once again, integration of services [51,52]: healthcare systems need to investigate the feasibility of providing antiretrovirals to pregnant women as part of routine maternal care in antenatal clinics. Task shifting and task sharing, which allow lower-level healthcare staff to assume greater responsibilities, could be of great assistance here [53]. Retention strategies are critical, too. Investigating the best means for maintaining contact with HIV-positive mothers and their infants is vital for PMTCT success. Family-centered care, which meets the continuing health needs of the entire household, may be a key strategy here [54]. Local programmes will have to learn how to adapt to their clients' cultural background, providing services that make them feel respected and safe.

### Uninfected infants exposed to antiretroviral drugs

Under the 2010 WHO guidelines, infants could have up to more than two years of *in utero *and post-partum antiretroviral drug exposure [3]. The immediate prophylactic benefits of this extended exposure are well recognized and outweigh any potential risks. There is, however, little knowledge of any adverse consequences for the resulting large numbers of HIV- and ARV-exposed but uninfected children, which is essential for optimal monitoring and management of any potential hazards.

Birth defects are an initial concern regarding first-trimester exposure to efavirenz. Studies conflict on this subject [55-57], and the WHO guidelines strongly advise non-pregnant women on efavirenz to use a secure form of birth control [3].

A number of studies have also reported conflicting findings regarding low birth weight and preterm deliveries following exposure to ARVs [58-61]. A 2007 meta-analysis found a large amount of variation in the different studies' methods and findings [62]. Overall, a greater risk of preterm delivery was associated with starting triple-drug therapy before pregnancy or in the first trimester and with the use of protease inhibitors at any point during pregnancy. An 8192-infant French study associated lower birth weights to triple-drug regimens compared with monotherapy, but inferred that the difference was due to confounding factors [61]. Chief among these was that pregnant women with advanced HIV in 1997-2004 were more likely than others to receive triple therapy. Resolving this nettlesome issue is essential for choosing PMTCT regimens and planning infant healthcare in areas of high HIV prevalence.

A further area of disagreement concerns mitochondrial toxicity in ARV-exposed, uninfected infants. Mitochondrial toxicity would lead to such conditions as lactic acidosis, pancreatitis, cardiomyopathy and neuropathy. Some cohort studies have observed signs of mitochondrial impairment in ARV-exposed infants [63,64]. Researchers have argued, too, that *in utero *HIV exposure itself leads to mitochondrial damage [64,65]. Yet another issue is haematologic toxicities: a longitudinal study of more than 4000 children aged 0 to 18 months found that haemoglobin levels, white blood cells and platelets were transiently reduced in ARV-exposed children [66]. Investigation is also continuing regarding effects of *in utero *ARVs on cardiac function and cardiomyopathy in HIV-exposed but uninfected infants [67].

Other ARV exposure concerns are an increased risk of infectious diseases in the newborn and potential for malignancies later in life [68,69]. There has been no confirmed elevation in malignancies, but cancers can take years to develop [70]. Longitudinal studies, including cohorts that follow up infants and children into adulthood, are required to answer this question.

As with malignancies, documentation of changes in growth and development has proved elusive. Here, too, studies have been carried out until only a few years of age [71,72]. More extensive and longer follow up is called for to detect subtle or lingering toxicities. Along those lines, the Paediatric HIV/AIDS Cohort Study in the United States has instituted a Surveillance Monitoring of Antiretroviral Toxicity project to prospectively follow more than 2000 ARV-exposed, uninfected infants to evaluate late toxicities [73].

The incidence of subtle adverse events is likely to rise as the length of ARV exposure grows as the revised WHO guidelines are implemented [3]. Studies of uninfected children will have to tease out the separate contributions of exposure to ARVs and maternal HIV and environmental and economic factors. Understanding these will be necessary to help identify the best strategies that minimize both the risk of HIV transmission and any compromise of growth and development. A more comprehensive pharmacovigilance system, for example, by expanding the existing network of antiretroviral pregnancy registries to include low- and middle-income paediatric cohorts, should explore models for longer-term monitoring of uninfected children exposed in pregnancy and during extended postpartum prophylaxis.

### New treatment options for children with HIV

The paediatric HIV incidence in high-income countries has shrunk to a very low level due to the success of PMTCT using suppressive triple-drug regimens. In resource-limited areas, where PMTCT coverage is suboptimal, an estimated 370,000 infants are born annually with HIV infection [74]. As with adults, WHO's 2010 paediatric guidelines will greatly broaden the goals for treating these children [2].

Studies have clearly demonstrated the survival benefits of providing immediate therapy to HIV-infected infants [75-77]. A groundbreaking South African study reported that deferring antiretroviral therapy until CD4 levels become low or symptoms appear increases infant mortality by more than 400% [78]. WHO, in response, recommended that all HIV-infected children under one year of age receive ARVs [76]. It is now raising that recommendation to the age of two years [9]. Recommendations for children older than two years include CD4 count and clinical criteria in line with those in the new adult guidelines.

Provision of immediate treatment to infants requires that they be identified in timely fashion. Postnatal follow up is notoriously weak in resource-constrained settings, and especially so for undiagnosed infants with HIV exposure [79]. Due to the persistence of maternal antibodies, infants younger than 18 months require sophisticated, expensive virologic testing for a definitive HIV diagnosis. Only 15% of infants born to HIV-positive mothers received such testing during the first two months of life in 54 countries reporting this information in 2009 [39]. The WHO guidelines include recommendations for symptom-based, presumptive diagnosis of severe HIV disease in areas where virologic testing is unavailable.

Strengthening infant services is critical to ensure prompt diagnosis and retention in HIV care. Integration of HIV care with other health services, along with task shifting and sharing, again promises to help ensure more consistent HIV management. Inclusion of community social networks and other household members (fathers, in particular) will provide substantial support for these efforts.

An issue particular to children older than age two years is the effect of ARVs on growth and development. If the drugs retard development, it would not be advisable to start ARVs earlier than necessary. The influence of HIV infection, however, confounds that of ARVs. Recent reports indicate that children who start treatment with less advanced HIV disease and less growth deficiency tend to gradually normalize their height and weight [80-82]. Children who initiate treatment later in the HIV disease process also gain weight and height, but at a slower rate. Even so, children remain on a growth trajectory determined by nutritional and environmental conditions. Analogous problems exist when studying other aspects of development, such as neurocognitive maturation and bone growth.

Starting ARVs also depends on the relationship between antiretroviral drugs and concurrent conditions. More research is required in this area, too. A particularly critical area is tuberculosis (TB) treatment in children. Mutual liver toxicities and drug-drug interactions create complicated treatment management problems for children infected with both HIV and TB. A study in South African children aged seven months to 3.9 years showed that it is possible to attenuate the tendency of lopinavir levels to decline in the presence of the TB drug, rifampicin, by using a higher dose of concomitant ritonavir (lopinavir/ritonavir ratio of 1:1 instead of the usual 4:1) [83]. The drawback is that children then have appreciable elevations in serum liver enzymes [84].

Paediatric treatment problems would be easier to resolve if there were as many available antiretroviral options as there are for adults. Unfortunately, many barriers retard development of paediatric ARV formulations for resource-restricted settings. Optimal and acceptable options for infants and children are of growing necessity because of concern about resistance due to single-dose nevirapine exposure during PMTCT, and also concern about emergence of resistance during treatment in children [85,86].

There is now low demand for paediatric ARVs in high-income countries, and so little financial incentive to bring new formulations to the market. In low- and middle-income countries, the available liquid paediatric formulations present a number of practical difficulties: bulk transport and storage is difficult because liquid formulations tend to have short shelf lives or require refrigeration. Also, their lack of palatability and large volume create household obstacles to adherence. The WHO guidelines stress that fixed-dose formulations combining multiple drugs in a single pill would be an important step to simplifying regimens [2]. Achieving advances of this order require multisectoral partnerships involving industry, private donors and international agencies [87].

### Moving forward

The Consensus Statement, "Asking the Right Questions: Advancing an HIV Research Agenda for Women and Children", issued by the IAS and its partners, identifies the research needed to bring advanced HIV treatment to women and children in resource-limited countries [9]. The statement's overarching recommendations call for more extensive HIV research on women and children, for widely sharing data, and for better dissemination of results. It makes a special appeal for consistently designing HIV research to include a provision for gender analysis. This analysis should especially stress significant parameters, such as retention in ARV programmes, sexual and reproductive health, morbidity and mortality, and loss to follow up (Additional file 1: Box 1).

The Consensus Statement includes several other major unmet research needs relevant to providing women with equitable, quality care. Scientific progress requires, first of all, designing observational cohorts and clinical trials to permit stratification by sex, race and ethnicity to provide the missing data needed to determine sex-based differences in pharmacokinetics and pharmacodynamics, treatment outcomes and adverse events (Additional file 1: Box 2). Well-designed, prospective impact evaluation studies could play an instrumental role in identifying new solutions as programmes are brought up to scale.

For children, the prime unmet need is a comprehensive system for tracking the continuing effects of HIV drugs. Expanding existing antiretroviral pregnancy registries to include low- and middle-income paediatric cohorts is an initial step. These cohorts should explore models for longer-term monitoring of uninfected children exposed *in utero *and during extended postpartum prophylaxis. The Consensus Statement further advocates advancing infant services to ensure prompt diagnosis and retention in HIV care. It also calls for a renewed research investment to develop improved paediatric ARV formulations, as well as advanced modalities for treating HIV and comorbid conditions in children (Additional file 1: Box 3).

Women and children alike would benefit from integration of HIV programmes with broader health services and a more family-centred approach. This possibility signals the need to intensify oft-ignored operations research on care delivery to identify strategies to optimize HIV treatment and PMTCT programmes for various populations. Special attention needs to be paid to linking women and children to a continuum of care - from HIV testing of pregnant women all the way through provision of PMTCT, early infant testing, and linkage to paediatric treatment and care as needed. The need is particularly critical for marginalized adolescents and adult women, women living in remote areas, single women, sex workers, women who use drugs, transgender individuals, indigenous women, and women from ethnic minorities.

Retention strategies are critical here. Investigating the best means for maintaining contact with infected mothers and their offspring is vital for successful HIV management. Local programmes will have to learn how to adapt to their clients' cultural backgrounds, providing services that make them feel respected and safe. Inclusion of community social networks and other household members, including men, may provide substantial support for these efforts.

As we summarize here, HIV research has a long way to go in addressing women and children's specific needs. Yet understanding their issues is central to controlling HIV, as well as other diseases. The research agenda laid out by the statement is designed to result in new strategic thinking for women and children, and supports a collaborative effort among key players, including those from industry.

## Competing interests

**QAK**, **CC**, **CH**, **EK **have no competing interests.

**AB **is an employee of Tibotec Pharmaceuticals Ltd. **PC **has served as: advisory board member in Avexa, Gilead, GSK, Myriad, Merck, Pfizer, Pharmasset, Schering Plough and Tibotec; Investigator in Avexa, Boehringer Ingelheim, Gilead, GSK, Roche, Merck, Pfizer, Pharmasset, Schering Plough, Tibotec, Abbott and BMS; Speaker (content and design performed by the speaker, no company control) for Abbott, BMS, Boehringer Ingelheim, GSK, Merck, Pfizer and Tibotec; and Scientific Advisor for Merck Sharp & Dohme, Pfizer, GSK, Avexa and Tibotec. He is not a shareholder in any pharmaceutical company; nor has he any commercial interest or investment in any pharmaceutical company. **MD **is an employee of Boehringer Ingelheim GmbH. **RD **is an employee of Abbott. **NH **is an employee of the Elizabeth Glaser Pediatric AIDS Foundation. **SL **is an employee of Merck. **JM **has received grants from, served as an *ad hoc *advisor to, or spoken at various events sponsored by, Abbott, Argos Therapeutics, Bioject Inc, Boehringer Ingelheim, BMS, Gilead Sciences, GlaxoSmithKline, Hoffmann-La Roche, Janssen-Ortho, Merck Frosst, Panacos, Pfizer, Schering, Serono Inc, Thera Technologies, Tibotec (J&J) and Trimeris. He is also supported: by the Ministry of Health Services and the Ministry of Healthy Living and Sport in the Province of British Columbia; through a Knowledge Translation Award from the Canadian Institutes of Health Research (CIHR); and through an Avant-Garde Award (No. 1DP1DA026182-01) from the National Institutes of Drug Abuse, at the US National Institutes of Health. He also received funding from Merck, Gilead and ViiV to support research into Treatment as Prevention. **SP **is an employee of ViiV Healthcare. **JR **is an employee and stockholder in Gilead Sciences. **RW **has affiliations as scientific advisor with Boehringer-Ingelheim, Gilead, GlaxoSmithKline, Merck, Pfizer, Tibotec, CareWorks, Togalabs, INSIGHT (NIH) and the International Partnership for Microbicides. **SH **is an employee of the International AIDS Society, and her salary is provided partly by unrestricted educational grants from the following pharmaceutical companies: Abbott, Boehringer Ingelheim, Gilead, Merck, Pfizer, Tibotec and ViiV Healthcare. For these authors, there are no conflicts of interest in co-authoring this article.

The conclusions and opinions expressed in this article are those of the authors and do not necessarily reflect those of their respective organizations.

## Authors' contributions

SH, with the support of independent consultant David Gilden, wrote the first draft. All authors contributed equally to the manuscript by providing comments on subsequent drafts. All authors have read and approved the final version.

## Pre-publication history

The pre-publication history for this paper can be accessed here:

http://www.biomedcentral.com/1471-2458/11/388/prepub

## Supplementary Material

Additional file 1 **Boxes**. Box 1: Overarching recommendations to improve research for women and children. Box 2: Recommendations for clinical and operations research for treatment for women and girls. Box 3: Clinical and operations research recommendations for PMTCT and paediatric care, treatment and support. Click here for file
