# Supplementary material for: Asking the right questions: developing evidence-based strategies for treating HIV in women and children
Source: BMC Public Health. 2011 May 25;11:388. doi: 10.1186/1471-2458-11-388 (PMC3118247; doi:10.1186/1471-2458-11-388)
Supplement: Additional file 1 — Boxes. Box 1: Overarching recommendations to improve research for women and children. Box 2: Recommendations for clinical and operations research for treatment for women and girls. Box 3: Clinical and operations research recommendations for PMTCT and paediatric care, treatment and support. [file 1471-2458-11-388-S1.DOC]

**Asking the right questions: developing evidence-based strategies for treating HIV in women and children**

**Additional files**

**Box 1**

**Consensus Statement:**

**“Asking the Right Questions: Advancing an HIV Research Agenda for Women and Children”** [Error: Reference source not found]

**Overarching recommendations to improve research for women and children**

- Invest in studies (e.g., prospective, retrospective and longitudinal) identified as optimal ways to answer the research questions outlined in this report.
- Data from existing operations research studies, programme evaluations and clinical trials should be more broadly shared, reviewed and analyzed to answer some of the specific knowledge gaps identified in this report.
- Research data should be disaggregated by sex to ensure opportunities for gender-based analysis using a variety of indicators, such as retention in ART programmes, morbidity and mortality, loss to follow up, and pharmacokinetic and pharmacodynamic parameters.

**Box 2**

**Consensus Statement:**

**“Asking the Right Questions: Advancing an HIV Research Agenda for Women and Children”** [Error: Reference source not found]

**Recommendations for clinical and operations research for treatment for women and girls**

**Clinical research:**

- Ensure clinical cohorts and clinical trial data are disaggregated by sex, ethnicity and race to support ongoing analysis of potential differences in pharmacokinetics and pharmacodynamics, treatment outcomes and adverse events in these populations.
- Establish appropriate studies to answer the questions about the impact and interactions of endogenous and exogenous hormones and antiretroviral therapy on health outcomes for girls and women.
- Assess the impact of antiretroviral drug prophylaxis to prevent inter-, peri- and post-partum HIV transmission on future antiretroviral therapy options and health outcomes for women.

**Operations research:**

- Conduct retrospective and prospective context-specific studies to identify how to leverage healthcare systems (including primary care, sexual and reproductive health services, prevention of mother to child transmission and harm-reduction/opioid-substitution programmes) to improve women’s access to and retention in healthcare.
- Conduct operations research, including programme evaluations, to improve antiretroviral therapy access, remove barriers to access and ensure long-term follow up for subpopulations of adolescent and adult women (e.g., women living in rural settings, single women, sex workers, women who use drugs, transgender individuals and women from different ethnic and socioeconomic populations).
- Conduct operations research, including programme evaluations, to identify nutritional supplementation interventions (micronutrient and macronutrient) for women that can be integrated into care, treatment and support programmes.

**Box 3**

**Consensus Statement:**

**“Asking the Right Questions: Advancing an HIV Research Agenda for Women and Children”** [Error: Reference source not found]

**Clinical and operations research recommendations for PMTCT and paediatric care, treatment and support**

**Clinical research:**

- Invest in innovative drug manufacturing and delivery systems (e.g., dissolvable films, microtablets) to address the need for appropriate paediatric formulations.
- Evaluate a range of weight-adjusted dosage recommendations and fixed-dose combinations for children.
- Invest in innovative TB and HIV diagnostics and monitoring to facilitate paediatric and early infant diagnosis and treatment.
- Review currently enrolled clinical trials and conduct necessary clinical studies to evaluate the impact of co-morbid conditions and their treatment on drug dosage and toxicity, with priority given to TB, malaria and malnutrition.
- Ensure a more comprehensive pharmacovigilance system by expanding the existing antiretroviral pregnancy registry to include low- and middle-income country cohorts/pilots and establishing an appropriate follow-up registry to evaluate the impact of ARV exposure *in utero* or during extended infant prophylaxis on uninfected children in resource-limited settings.
- Review existing data and currently enrolled trials to establish optimal treatment strategies for children.

**Operations research*:**

- Review existing data and conduct studies to determine the most effective strategies for providing and monitoring CD4 testing and ART for treatment-eligible pregnant and breastfeeding women.
- Review existing data and conduct studies to determine the most effective implementation strategies for prophylaxis during breastfeeding and the comparative effectiveness of infant versus maternal prophylaxis.
- Review existing data and conduct studies to determine the feasibility and impact of providing ART for eligible pregnant women within routine maternal and child health services in antenatal clinics.
- Review existing data and conduct studies to determine the effect and impact of task shifting on PMTCT and paediatric care, support and treatment scale up in various settings, at various levels of the healthcare system and among different cadres of health workers.
- Review existing data and conduct studies to determine what interventions at the programme, facility, community and household levels have the greatest impact on retention in paediatric HIV care, especially in the first 12 months of life.

* Questions in this research category in the Consensus Statement – operations/implementation research related to PMTCT, including paediatric care, treatment and support – were addressed by a parallel consultative process led by UNICEF. That initiative involved a high-level consultation with the Interagency Task Team for PMTCT including the WHO, UNAIDS and US agencies in collaboration with George Washington University and the Elizabeth Glaser Pediatric AIDS Foundation.
